# Driven by Vision: Learning Navigation by Visual Localization and Trajectory Prediction

**DOI:** 10.3390/s21030852

**Published:** 2021-01-27

**Authors:** Marius Leordeanu, Iulia Paraicu

**Affiliations:** 1 Institute of Mathematics of the Romanian Academy (IMAR), Calea Grivitei 21, 010702 Bucharest, Romania; iulia.paraicu@stud.acs.upb.ro; 2 Department of Automation and Computer Science, University “Politehnica” of Bucharest (UPB), Splaiul Independenței 313, 060042 Bucharest, Romania

**Keywords:** autonomous driving, self-driving, visual localization, visual navigation, deep learning, trajectory prediction, geometric computer vision, autonomous driving dataset, localization by image segmentation

## Abstract

When driving, people make decisions based on current traffic as well as their desired route. They have a mental map of known routes and are often able to navigate without needing directions. Current published self-driving models improve their performances when using additional GPS information. Here we aim to push forward self-driving research and perform route planning even in the complete absence of GPS at inference time. Our system learns to predict in real-time vehicle’s current location and future trajectory, on a known map, given only the raw video stream and the final destination. Trajectories consist of instant steering commands that depend on present traffic, as well as longer-term navigation decisions towards a specific destination. Along with our novel proposed approach to localization and navigation from visual data, we also introduce a novel large dataset in an urban environment, which consists of video and GPS streams collected with a smartphone while driving. The GPS is automatically processed to obtain supervision labels and to create an analytical representation of the traversed map. In tests, our solution outperforms published state of the art methods on visual localization and steering and provides reliable navigation assistance between any two known locations. We also show that our system can adapt to short and long-term changes in weather conditions or the structure of the urban environment. We make the entire dataset and the code publicly available.

## 1. Introduction

Nowadays, self-driving cars and intelligent driver-assistant systems heavily relying on vision are emerging in our everyday reality. Still, the complex task of learning to navigate only from visual data is in its early stages. Even though we acknowledge that for self-driving cars, additional control modules are necessary (such as the ones based on GPS and LiDAR), it is essential to increase the performances and the perception of the vision-based solutions. Since vision, at both short and long-range distances, is almost always available at relatively low-cost.

In the literature, there are models [1,2] that use visual information to extract high-level semantics of the traffic scene and decide the steering action conditioned on these representations. Other works [3,4,5] propose end-to-end models that take as input, frames from the video, and directly output steering commands. The first approach is easier to interpret by humans, being useful to identify and justify failure cases. On the other hand, collecting data and training is more efficient in end-to-end solutions, which can learn more relevant features.

**Figure 1:**
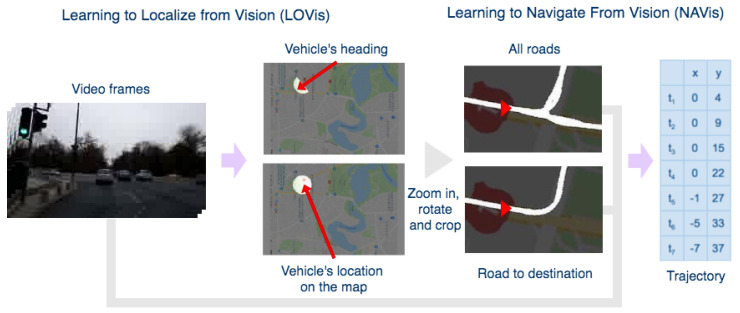
The high-level structure of the system. The first ConvNet module learns to predict the location and heading on the map by an image segmentation-based approach. Road map segments are cropped around the location, one showing all directions, and the other one only the intended route, conditioned on the destination. From road map crops and video frames, the second model predicts the navigation trajectory for the next seven seconds, which is also conditioned on the final destination. The trajectory estimation task becomes very interesting and useful especially in intersections with several possible routes.

We propose a system (Figure 1) that combines end-to-end learning with precise mathematical modeling for automatic visual localization and navigation, thus providing both efficiency and a certain level of explainability. Our model predicts the vehicle’s trajectory for the next seven seconds, which offers complete steering and speed information to avoid obstacles and follow a certain route. Our work is thus related to end-to-end learning approaches, which started with the pioneering model Alvinn [6] and continued with the highly successful models in the deep learning era [3,4,5,7]. 

### 1.1. Differences between Visual-Based and GPS-Based Localization and Navigation

There are several important differences and advantages that a visual-based navigation system could have over a more traditional GPS-based approach. The two, in fact, have complementary properties and could be used in combination in a practical system, for the benefit of the overall accuracy, speed and robustness to various challenging road conditions. Below we discuss some of these aspects, while emphasizing the advantages that visual-based navigation could bring:

1. Visual-based navigation systems, using deep convolutional nets, could respond faster than GPS-based navigation systems since deep nets offer a local, immediate and direct mapping between visual input and the desired localization and navigation output, without needing external signal from satellites and various, often expensive, search and other algorithmic procedures. It is well known that current GPS-based commercial navigation applications can have a significant delay (often up to a second), which could make driving very difficult and even unsafe. It is not unusual for a regular driver to miss, for example, an intersection due to such a delay.
2. Visual-based systems could also be more accurate than GPS-based systems, since vision, which is based on local sensing information, can offer a vehicle pose (position and orientation) that is better aligned with the real scene. At rest or very low speeds it is well known that GPS cannot offer such accurate orientation.
3. GPS signal is not always available or could be erroneous, especially in cluttered urban or natural areas, with many tall buildings, trees and other large structures, or under bridges, tunnels and structures that could occlude the sky. GPS could in fact fail for many other reasons, in which case a visual based navigation system could take its place.
4. In general, we could think of GPS-based and vision-based navigation as having complementary properties such that in practice they could work together, benefiting from each other’s advantages. While GPS is robust to weather conditions and traffic and does not need prior learning, the visual system could be faster, sometimes even more accurate (especially in areas cluttered by tall structures) and it could also estimate orientation correctly even in cases of zero or very low speed. Together, GPS and visual-based localization and navigation, could form a more robust and more accurate system. This hybrid combination between GPS and vision is definitely worth becoming the subject of future research. In this paper we focus on vision only, in order to better understand its capabilities and limitations.

**Recovering from errors in visual-based localization and navigation:** A visual-based navigation system that involves significant temporal processing could in principle be vulnerable to past errors, which could also accumulate over time or cause the system to suddenly drift to a completely new and distant location. However, in the case of the Driven by Vision system presented here, we recover from errors easily since the system does not depend on past predictions. All estimations are done on a small number of frames around the current position and time.

### 1.2. Visual-Based Localization

One of the main tasks we tackle is that of visual-based localization, which aims to estimate location from visual input (e.g., images or video). Traditional methods performing this task were based on explicit feature extraction and matching [8,9]. As with the vast majority of vision problems, the accuracy in localization raised considerably with the advances in deep learning [10,11,12,13,14]. Feature-based methods are highly accurate but often require complex pipelines and are not very robust to changes in weather, lighting conditions, and occlusions.

Kendall et al. [14,15] introduced the idea of predicting the observer’s pose directly from the image with an end-to-end regression CNN. They also use transfer learning from classification nets trained on large datasets such as Places and ImageNet. In their remarkable work, the model achieves good accuracy in real-time, being robust to factors such as unusual lighting or image blur.

Marcu et al. [12] proposed an original deep-learning approach to localization from images that formulates localization as a segmentation task: the input to a segmentation-style U-Net [16] is a given image and the output is a circle on a map, with the center at the predicted location of that image w.r.t. the map. We compare these methods on our dataset, then propose an improved localization by segmentation solution that achieves strongly superior results to the regression paradigm.

### 1.3. Visual Navigation with Location Information and Trajectory Prediction

The large-scale usage of navigation applications such as Google Maps or Waze makes it possible to provide both directions and global traffic dynamics. Some driving assistance solutions [17,18] even generate videos from Google Maps images to offer better route recognition to users.

In the context of autonomous driving, Hecker et al. [7] introduced the idea of training end-to-end models with additional online information about the navigation route. Their deep recurrent CNN model receives images from multiple cameras placed at different angles along with a screenshot of a route planning commercial application. Amini et al. [19] propose a variational end-to-end solution for navigation and localization that predicts the probability distribution of the vehicle’s next pose given an offline map representation, the previous pose, video, and GPS raw streams.

Another task we tackle is that of trajectory prediction. The idea was firstly introduced by Glassner et al. [20], who developed a trajectory learning model that exceeds a baseline end-to-end steering solution in a simulated highway environment. Their neural network is followed by an analytical module designed to validate the predicted trajectories. Different from the model in [20], ours predicts trajectories for a longer time span and is trained and tested on real-world data. The urban scene, with intersections where there are multiple possible paths to take, increases both utility and complexity of our task. Apart from comprising rich steering information and being more natural to interpret, trajectories can also be fitted in the post-processing step to derive smoother steering actions and improve passengers’ comfort (studied in [21]). At the higher level, we aim to solve visual-based real-world navigation, by estimating the trajectory without any GPS knowledge, conditioned on the desired destination relative to a map (automatically created during training). Our approach, based solely on visual information in order to perform navigation is different and complementary to most systems which involve GPS information for autonomous navigation [22,23,24,25,26,27].

Mirowski et al. [28] proposed the work that best captures the learning of visual navigation with a reinforcement learning approach. They solve the maze navigation task in a context closer to real life, by also integrating it with place recognition, which we exploit as well. Their agents successfully learn to reach destinations by taking discrete movement actions in an environment made of Google Street View images. While our learning approach, automatically supervised by GPS signal during training, is very different in terms of data, learning models, mathematical formulation, and specific predictions (our trajectory vs. their discrete set of actions), our work and theirs relate at a high level.

### 1.4. Robust Visual Solutions in Changing Conditions

Environmental changes such as weather, day-night and long-term structural changes over time, severely impact the performances of visual models performing various tasks. Recent research [29] addresses the impact of such factors on the accuracy of visual localization models. They also introduce three datasets for this purpose and benchmark the current localization methods, concluding that we are far from having visual localization robust to time changes. Some approaches [30] tackle this problem by using additional semantic information and inserting a new step in a feature-based localization method pipeline. Even though their solution is effective for some use cases, such semantic information is not available in our setup and would be expensive to collect.

Several real-world self-driving datasets [4,5,7] also include frames having challenging luminosity or weather conditions, as well as ours. Still, they only represent a small percentage of the test data and the authors present only the general, average results of their methods over all cases. Only very recently researchers [31,32] proposed advanced artificial weather generation techniques, for augmenting real world datasets (KITTI [33]) with multiple weather conditions. Their models trained on such data perform considerably better on real bad weather (taken from other datasets).

In experiments (Section 6.4)), we show that by training on an extended dataset, which includes images with various changes in weather, under diverse conditions of lighting, sun flare, rain and fog, as well as structural and environmental changes that take place over a longer period of time (14 months), the results on the test set improved. Therefore, the addition of such diverse conditions and environmental changes improves generalization.

## 2. Motivation and System Overview

Our main goal is to replicate humans’ capacity to navigate from vision alone with minimal expenses. A robust visual navigation solution could also complement the current GPS based ones by removing the localization delay, by knowing orientation without needing movement and by understanding fine traffic dynamics. Figure 1 presents an overview of the system.

**Figure 2:**
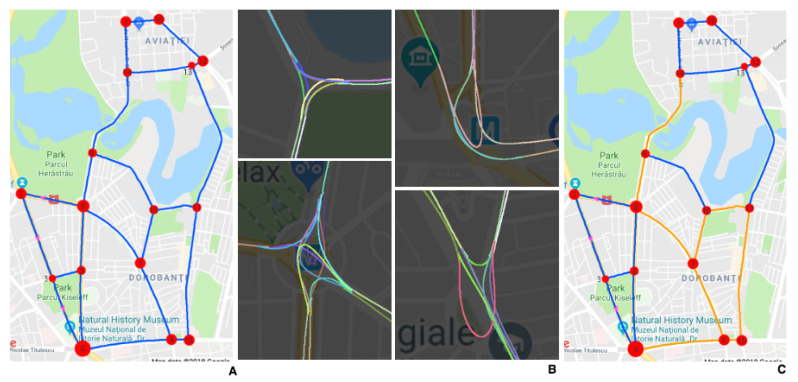
(**A**) The graph structure of the driving map. (**B**) Cropped sections of the analytically obtained map overlapped with the corresponding map regions from Google Maps. (**C**) The sub-part (orange) of the map crossed in the second phase of data collection.

In the first stage, a deep net predicts from the current frame the corresponding 2D pose w.r.t to a known map. In the second stage, the route around the obtained location is fed together with multi-frame visual information to another deep net, which learns end-to-end to predict the future trajectory. Due to the lack of data freely available that is suitable for our proposed approach, we collected our own dataset covering a relatively large area in a European city (Figure 2).

We make all our data and models freely available on our website Driven by Vision project website: https://sites.google.com/view/drivenbyvision/home). Our setup is simple and inexpensive: we use a regular car and a mobile phone that collects video and GPS streams simultaneously. The ground-truth location and steering labels for the video frames are generated without human annotation, by automatically processing the GPS stream, thus obtaining annotations at a minimal cost. A system such as the one we propose could be easily deployed within a city, for both data collection, annotation, training, and driving assistance, as it requires no specific hardware or manual annotations.

### Main Contributions

The main contributions of our proposed approach are:

1. We introduce, to our best knowledge, the first deep-learning-based system that simultaneously learns to self-locate and to navigate towards a planned destination from visual information only. This is an important ability and complementary to the case when GPS information is available. Due to the loss or inaccuracies in GPS signal, often met in practice, the capacity to accurately locate using visual information can significantly improve the performance and robustness of current GPS-based methods.
2. The system is highly scalable at minimal costs and can be easily deployed to learn over an entire city or other geographical region by having it used by many drivers simultaneously. We also introduce the Urban European Driving Dataset (UED), which we make available along with our code and user-friendly application, which we developed for both data collection and real-time usage.
3. We present competitive numerical results, improving over strong baselines and state-of-the-art methods.
4. Other contributions: (1) we extend and improve a previous visual localization by image segmentation model and adapt it to learn to localize accurately in challenging traffic conditions; (2) we output trajectories, functions of space vs. time, which comprise steering and speed information for up to seven seconds in the future; (3) the map is created analytically and automatically from the collected GPS data, with no human intervention; (4) we make the localization and navigation components robust to problem-specific noise.

## 3. Creating the Urban European Driving (UED) Dataset and the Map

We used a mobile phone to film through the windscreen of the car while driving. A GPS stream was collected simultaneously with the video by using our custom Android application. The map representation (Figure 2A) of the roads covered by the dataset is a directed graph where nodes and edges represent intersections and roads between intersections, respectively. The resulting graph contains 16 nodes and 41 edges, with a total length of 35 km.

The dataset contains two parts: one collected in late autumn (91.23%) and the other one after 14 months, in mid-winter (8.77%). We used the first part data in the main experiments (Section 6.2 and Section 6.3), and the second part to evaluate the methods in different environmental conditions (Section 6.4).

For the first part, we chose the dataset routes by a simulation designed to cover the graph in a manner that is both uniform and realistic. From the current node, it generates the shortest route to a random destination, and then the process is repeated with the previous destination node as the source until a limit of 350 km is reached. The simulation was run 1000 times, and the best candidate was selected to be the one with the minimum number of pair-edges (two oriented edges connected by a node) that are crossed less than three times. We maximize pair edge crosses because they define the actual route, as an intersection can be passed through in different ways depending on the destination.

In the second collection phase, we select a continuous circuit from the map (Figure 2C) and cross it four times, in separate days. The UED Dataset consists of 21 h and 5 m of driving videos at 30 fps.

### 3.1. Exploring the Urban European Dataset (UED)

The main goal of our approach is to be able to learn localization and navigation from visual information, with minimal costs for data acquisition and labeling. This makes the proposed system suitable for real-world applications, overcoming the actual limitations of current GPS-based system and being easily adaptable to work in conjunction with those, for improved localization and navigation. Our automatic solution for data collection and annotation are therefore suited for this purpose, resulting in a unique dataset, which is indeed complementary to the already existing ones.

Table 1 shows a comparison of published driving datasets, which are dedicated to self-driving tasks that are related to ours. As shown in the table, our UED dataset is of medium-to-large size among the others. However as the dataset setup is accessible, so it can be easily scaled, the same way as in [5]. Even though BDDV [5] was collected in the same low-cost, scalable manner, it provides no information about the driver’s route, which is essential for navigation. We choose to process the GPS stream to obtain steering labels, instead of using the driver maneuvers read from the vehicle’s CAN bus. Using a CAN bus reader, as well as lidar, or specialized cameras would make the acquisition process more expensive and harder to scale. Hecker et al. [7] have shown that route planners can improve learning to drive by also providing knowledge about the destination. Here we push this idea further into the visual world and create an autonomous route planner based on our visual localization module. In conclusion, we introduce the first dataset in the literature that has self-generated route information from a module that is GPS independent at test time.

**Figure 3:**
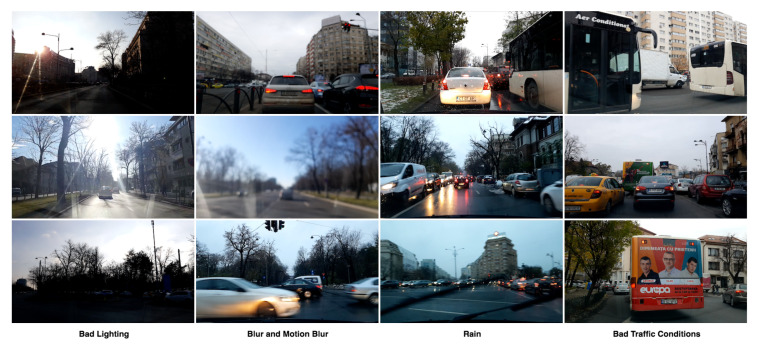
Samples from the dataset taken in challenging conditions such as bad lighting, blur, rain, and heavy traffic.

In Figure 3, we show diverse environmental conditions from our dataset. The bad lighting caused by the undesired position of the sun with respect to the camera is a common condition. Also, heavy traffic, causing large occlusions, is often present in the videos. The weather conditions across the dataset vary, including light rain and snow. Some frames are affected by blur caused by camera motion, object motion, or camera losing focus. Therefore, we propose a diverse dataset, being the first one having self-generated route information, and which is highly scalable.

**Comparison between our UED dataset and current real-world driving datasets:** In Table 1, we compare several real-world datasets, including ours, along several relevant dimensions such as: the driving time, the availability of GPS signal, Lidar information, accessible setup (so it could be easily used by other reasearch groups), availability of its own route planner (vs. the usage of a commercial application for route planning). For example, our navigation model learns trajectories conditioned by the destination. To achieve this, it receives route information along with the video frames. Unlike [7], which uses a commercial application for route planning, we create an autonomous route planer, based on the visual-localization module and the self-created analytical map. To keep the data collection as simple as possible, our UED dataset offers vehicle steering labels derived from GPS as opposed to using CAN bus reader. We choose a highly accessible setup for collecting data, using only a regular car and a smartphone, to be able to extend the current dataset easily (similarly to [5]). That is why we avoid on purpose using specific or high-cost equipment such as lidar, CAN bus reader, and dedicated video cameras. From the information given in Table 1, one can see that our dataset as several important practical advantages over the current available datasets in the literature, which could facilitate further research in the community.

### 3.2. Polynomial-Based Analytical Techniques for Modeling Paths, Trajectories and Creating the Map

Estimating the vehicle trajectory (position as function of time) is at the core of our navigation system, as shown in Section 5. Here we present a general polynomial-based trajectory fitting method, which we use for estimating such trajectories. The same basic mathematical model and approach is then immediately adapted to estimating vehicle paths (positions as functions of distance travelled), which is at the core of computing an analytical model of the map, as shown in the next Section 3.3.

Due to the large data space and noise in the data, ConvNets sometimes express undesired and unpredictable behavior. Precise mathematical models can often complement neural nets (NN), to reduce such problems and improve generalization. In this paper, we employ an analytical approach based on polynomial functions of time or distance, used for fitting time trajectories (functions of time) and map segments (paths—functions of distance), respectively. We chose the mathematical model of polynomials for trajectory fitting because polynomials are flexible and also relatively easy to estimate and compute [38]. Given the initial 2D trajectory points, each trajectory can be analytically modeled with two polynomial functions of time for the *x*, respectively *y* space components. For simplicity, we exemplify here only the 3rd order case, for which we solve for the polynomial coefficients. The 3rd order case immediately generalizes to higher orders (in our actual implementation we used 5-th order polynomials):

(1) $$\begin{array}{cc} x(t) & =a_{1}t^{3}+a_{2}t^{2}+a_{3}t+a_{4} \end{array}$$

(2) $$\begin{array}{cc} y(t) & =b_{1}t^{3}+b_{2}t^{2}+b_{3}t+b_{4}. \end{array}$$

As the x-y coordinates are known along with their time steps, the coefficients can be estimated using the method of linear least squares, by forming the data matrix T, with values $t_{i}^{3},\;t_{i}^{2},\;t_{i}$ on the i-th row and the x and y vectors containing the target $x(t_{i})$ and $y(t_{i})$ values. Then the optimal coefficient vectors a and b of the convex least squares problem are found using the classic formula: $a={\left(T^{T}T\right)}^{-1}T^{T}x$ and $b={\left(T^{T}T\right)}^{-1}T^{T}y$. After obtaining the polynomial coefficients, we can analytically find x(t) and y(t) for any time step *t*. The analytical approach ensures that the resulting trajectories are smooth and makes it possible to sample XY points in the future at equal time intervals.

### 3.3. Analytical Map Representation Using Polynomial Path Fitting

Visual map representations can improve the prediction of a driving neural net, especially in intersections, as shown in [7,19,21], who provide their models with visual map captions around the current GPS location. Different from them, we analytically derive the map from the collected GPS streams of the routes traversed in the training phase.

The key idea in map creation is fitting polynomials on conveniently chosen compact groups of location samples to obtain curved map segments that together form the entire map. We define the map segments as follows: (1) each directed edge of the graph (connecting road) is a segment. (2) nodes (intersections) include multiple segments. They connect each pair of distinct edges with direction inside the node. The map creation algorithm takes as input lists of geolocation samples, nodes centers’ coordinates, and their radius. It also implies that every two consecutive map segments along a route continue one into the other smoothly. The steps for building the map representation are:

1. Place each geo-coordinate sample into its segment bucket together with its distance to the start of the segment. The distance to the segment start is 0 when entering the segment. Then for each following sample point, its associated distance is the previous point distance summed with the euclidean distance between the two samples.
2. Fit polynomial functions of distance for each segment given the points and the corresponding distances in its bucket, using the method presented in Section 3.2, but using x(d) and y(d) instead of x(t) and y(t), where *d* is the known distance to the start of the segment. The degree of polynomials is directly proportional to the length of the modeled segments but significantly smaller than the number of points.
3. Using the analytical model, we sample points at 1 m distance interval along each segment from s=0 to the segment’s end. After this step, some pairs of segments (consisting of the sampled points) will not connect smoothly, having small gaps in between. To tackle this, for each segment we refit its polynomial function, by considering the ending points of its neighboring map segments, and then sample from s=−delta to s=end+delta (delta is a small distance buffer) to obtain a final smooth map representation.

**Further understanding of the analytical map:** In Figure 2, part B, we present cropped sections of the analytical map, with different path segments (each connecting two intersections/nodes), shown in different colors, fitted as higher order polynomial curves. These polynomials are functions of distance between their start and end intersections (nodes). We make sure that the path segments that meet at a given intersection (the end of the incoming one meeting the start of an outgoing one) smoothly continue one into another by using a common, small overlapping subset of x-y locations (right at the place where they meet), when we fit them using least squares. The cropped analytical map examples in Figure 2, part B, also show how complex the map could become in intersections where many roads enter and exit. These situations further justify our visual trajectory prediction approach, in such complex cases where there are many ways to approach and exist a large intersection (changing lanes and direction, avoiding obstacles and other cars in traffic), depending on the entrance point and the destination.

## 4. LOVis: Learning to Localize from Vision

Our localization from vision system (LOVis) has to recognize the current location of the automobile from a continuous online video stream. We test two previously proposed approaches, one formulating localization as regression and the other as segmentation, then extend the last one and obtain significantly better performances.

### 4.1. Localization by Regression

In [14,15], authors train a regression NN to predict the 6 degrees of freedom (DOF) camera pose from single images of landmarks. The model’s output Urban European Dataset (con) two vectors: the 3-component position vector of distances on the axes *x*, *y*, and *z*, and the 4-component quaternion vector of rotation around the three axes. The loss function is the weighted sum of the L2 norms between each of the outputted vectors and their corresponding ground-truth. In [14], the weighting is done by hyper-parameters, whereas in [15] the weights are learned based on the homoscedastic uncertainty of tasks [39]. To express the vehicle’s pose on a 2D map, we only need a 3-DOF representation. When implementing the models above, we keep the same setting and evaluate only the pose components of interest.

### 4.2. Localization by an Image Segmentation Approach

We first introduced the idea of learning localization by segmentation in [12] for the case of satellite images with associated geolocation (2-DOF pose). The two-stage model predicts the mask of a dot on an output map (representing the geographic map), such that the center (*x*,*y*) of the dot represents the coordinates of the image location w.r.t to the geographic map. Treating geolocalization as segmentation has the advantage of capturing well the relation between “what” and “where”, between semantics and the geometry of the output space. Also, in the case of complex output distributions, a segmentation net can predict several possible locations (e.g., output several dots, later post-processed for getting a final answer), whereas regression nets can only produce a single output.

**Figure 4:**
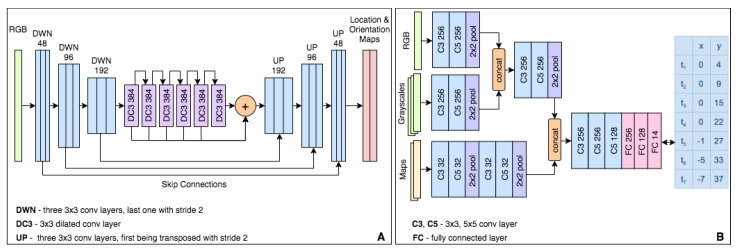
The network architectures we propose for localization (**A**) and navigation (**B**). The localization module **A** outputs the vehicle 2D pose (location and orientation) using a segmentation representation (a dot on the map for localization, and a half dot for orientation). The navigation module (**B**) outputs the predicted future trajectory (next seven seconds) by seven 2D points, one per second in the future.

The second stage module in [12] is the basis for our architecture. We extend the network in two distinct ways to predict 3-DOF poses end-to-end from RGB input images. One approach (LOVis-reg) is by adding a regression branch after the last encoding layer, which outputs the value of the observer’s camera orientation. The other full-segmentation approach (LOVis, Figure 1 and Figure 4A) outputs a secondary orientation map, on which only half of the dot is segmented towards the vehicle’s heading direction. The orientation is obtained from the vector connecting the center of the dot (on the first map) with the center of the half-dot (on the second map).

### 4.3. A Deeper Look into the Localization by Segmentation Model

We analyzed the failure cases for location prediction, when the net makes large errors or does not segment any region. Most such cases are on consecutive frames that contain occlusion elements, like large vehicles right in front of the car.

For usual segmentation tasks, the segmented area also corresponds to the relevant region in the input image. This observation does not apply to the localization task, which has no direct logical relation between features’ positions in the input and the position of the dot in the output map. However, there are methods [40,41] in the literature that discover which locations in the visual input are relevant for a particular network’s output response, based on the activation values of the neurons in forward and backward passes. The guided backpropagation [40] algorithm fits best our case, as it outputs a fine-grained relevance map of the input image, and it works for any CNN.

**Figure 5:**
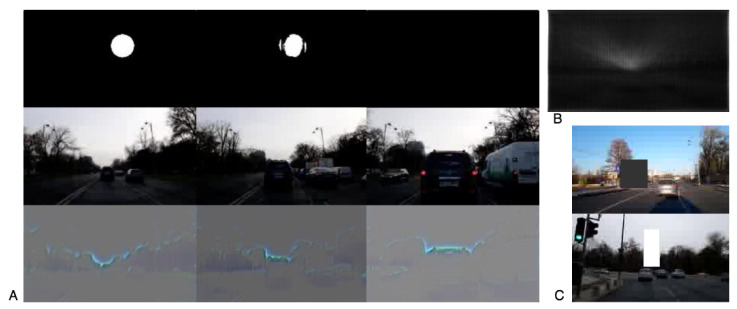
Analyzing the network’s attention by applying the guided backpropagation technique. (**A**) The top row shows the prediction of the network for the observer’s location, while the bottom one shows the visual cues of most interest for the network. It can be seen that as the camera approaches the SUV vehicle, the skyline shape changes and the segmentation of the dot fades away. (**B**) The guided backpropagation mean pixel activation values over the training set images. (**C**) Augmented images (with occlusions being added in the form of rectangles of constant grey level) used for robust training.

Let us take a closer look at the case presented in Figure 5. When the camera is far from the vehicle in front (images shown in the middle row in Figure 5A), the localization works fine and a dot appears on the localization map. However, the moment the vehicle in front gets very close to the camera, the dot disappears. The saliency map obtained by guided backpropagation clearly shows that the model’s attention is on the contour of the skyline, with a higher weight on the central region of the image (Figure 5A). Therefore, when a taller vehicle comes very close to the camera (as it is the case in the figure) it obstructs the parts of the skyline which are relevant for localization, so the output immediately degrades: the segmented dot loses its shape, it is miss-placed or even disappears (as it happens in the case presented in Figure 5).

In order to avoid this phenomenon, we must consider visual parts of the image that are less affected by such distractors. For this purpose, we introduce an informed technique for augmenting the training data. Firstly, the mean of each input pixel’s (guided backpropagation) activation values is computed (map shown in Figure 5B). From all calculated values over the training set, an empirical distribution map is obtained. Thus, positions in the image corresponding to pixels with higher probabilities are more likely to influence the network’s predictions. Then, for each training image, we randomly sample from the distribution a pixel location in which we center a randomly sized box of constant random gray-scale value (to mimic a large vehicle in front of the camera). This procedure is exemplified in Figure 5C.

## 5. NAVis: Learning to Navigate from Vision

The final stage of our method uses the analytical map and the visual localization net, to learn how to navigate in dynamic traffic between previously seen places. We predict trajectories to train a model (NAVis) that is better capable of following a route. Also, it produces significantly fewer high-frequency oscillations and local errors than models with simple steering output.

The navigation net architecture we propose is shown in Figure 4B). The inputs to the network come along three branches: one is for the current RGB frame, another one for three grayscale frames sampled uniformly from the last two seconds, and the third one for two binary images of the analytical road map. The first road map contains all roads around the current location, while the second one only shows the road segments that are part of the vehicle’s route. The width of a pixel in the map images represents 1 m in the real world. The map is limited to a local neighborhood centered at the predicted vehicle location. The model outputs seven pairs of real (x,y) values corresponding to points coordinates defining the trajectory, one per second. The training loss function *L* is the mean of the euclidean distances between predicted and truth points (N=7):

(3) $$\begin{array}{cc} L & =\frac{\sum_{n=1}^{N}\sqrt{{\left(x_{n}^{t}-x_{n}^{p}\right)}^{2}+{\left(y_{n}^{t}-y_{n}^{p}\right)}^{2}}}{N}, \end{array}$$

where $\left(x_{n}^{t},y_{n}^{t}\right)$ is the location of the n-th ground truth point on the real trajectory (as driven by a human pilot on a given trajectory training case) and $\left(x_{n}^{p},y_{n}^{p}\right)$ is the predicted location of the NAVis network for the same n-th point. There are seven points, one for each second in the future, up to seven seconds. The loss is the L2 distance between the two vectors of seven points, the predicted and the ground truth one, respectively. The loss presented in Equation (3) is, of course, given for a single training case. Then, the overall training loss is the sum of all such L2 losses for all trajectory training cases.

### A Deeper Look at the Visual Navigation Capabilities of the System

Now it is time to discuss in more detail the actual navigation capability of our system in practice, for a better understanding of our trajectory prediction module (NAVis) and how it can help in improving navigation. Once the position and orientation of the vehicle is found by the localization LOVis system, we know where we are on the map, which has been automatically learned and stored (Section 3.3).

The map is like a graph with nodes being connected by path segments. Once the current pose is known and the destination point is given, the optimal route between the start node and the end node in the map graph is easily found algorithmically, by the classic Dijkstra’s method. The global optimal route is instrumental in knowing, at every point on the map, which is the previous intersection (A: start node of the current path segment/edge), the next intersection (B: end node of the current edge) and the following intersection C, which follows immediately after the next one B. This intersection C can be found only by knowing the optimal global route. Determining the intersection C that follows the next one (B), is conditioned on the final destination, that is why we need to compute the optimal route first.

Once the three nodes (A,B,C) are known (the previous (A), the next one (B) and the one after the next one (C)), we can immediately show to the trajectory prediction network only the part of the local map around the next intersection (B), which is restricted to the correct route (going from A, through B, towards C). This local map, showing only roads along the correct route around the next intersection B, becomes one of the inputs to the navigation deep net (see Figure 4B). Thus, the map, restricted to the correct route, is crucial in predicting the correct trajectory once we enter the next intersection B.

The role of the visual navigation module is then to show the optimal trajectory in the next seven seconds towards the desired destination. Different from the classic GPS-based navigation systems, we consider not only the optimal global route (on an abstract map graph) but also the actual traffic and complex situations which might arise in complex, large intersections, where lanes need to be changed and other vehicles need to be avoided in a proper manner. While a GPS-based system is not considering the current visual input from the local, actual scene, our predicted trajectory does consider such input, which changes dynamically, on the spot.

Being blind to what is going on in the actual scene, a standard GPS-based systems cannot adapt and take in consideration the continuously changing information that comes from traffic and the exact location and orientation of the vehicle. The delays in the GPS-based system, slight errors in GPS localization and orientation, lack of information about the traffic and the absence of the exact visual field of the pilot, make it impossible for a GPS-based system to predict accurate and safe short-term trajectories. However, such trajectory prediction is important for reaching the final destination, especially in the complex cases of heavy traffic and large intersections, for which a classic GPS navigation system becomes difficult to use.

Thus, our Driven by Vision system not only that it shows which is the best trajectory based on the best global optimal route to destination, but it also takes in consideration the cars in front, which it tries to avoid, and the exact position on the road (the lane in which the car is), when it predicts such trajectory. Also note that such trajectory, which is represented as a function of time, automatically provides speed, acceleration and braking information, as well as steering information for the next seven seconds (as first and second order derivatives w.r.t time).

Therefore, in some sense, our visual-based navigation system, while important for good and safe navigation, is more local than the larger, global navigation problem in the graph (which we perform using classic graph search techniques). At the same time, our navigation is more global than a very simple steering angle prediction (which is the main estimation task for autonomous driving in the current vision literature). In Section 6.1 we explain in more detail how the evaluation of the trajectory prediction is performed, in terms of both lower level error distances as well as the higher level of navigation.

For a better understanding of the functioning of our localization and navigation system we provide several video demos on our Driven by Vision project website: https://sites.google.com/view/drivenbyvision/home). The demos offer a clearer picture of how our Driven by Vision system works.

## 6. Experimental Analysis

In the next section we discuss and motivate the methodology we chose for our experimental analysis and the scope of the experiments.

### 6.1. Methodology of Experimental Analysis

We organize the experiments in three main categories, along different tasks.

**Planned experimental analysis of localization:** First we plan to test the capabilities of the visual localization module (Section 6.2) and compare it to published top methods in the field, which rely on the exact same type of visual information (Table 2). Most methods in computer vision on visual localization treat the task as a regression problem, with the pose being predicted as a set of real numbers. Our extensive tests show that the approach in which we treat localization as image segmentation (initially introduced in our previous work [12]), is superior to regression-based approaches. Therefore, one of our goals here is to compare the case of treating localization as regression vs. the case of posing it as a segmentation task.

While segmentation is in general more accurate than regression, one of its weaknesses could be that the localization response rate (the rate at which an actual dot appears on the localization map) might not always be 100%—which means that an actual localization is not always given. This could happen especially when the input is not of good quality and the localization deep net has low confidence in the accuracy of prediction. On the contrary, the regression case always provides an output, even when such output could be completely wrong. Thus, another key aspect to analyse is the response rate of the different methods compared, where the regression case will always have a response rate of 100% (Table 2).

Next, once a location prediction response is given, we look at two types of errors, the mean (average) and the median errors in meters. The median error is very relevant since the presence of outliers strongly affects the mean error. However, outliers could be, in principle, detected, using various robust techniques, including RANSAC (Random Sample Consensus) algorithm (for line or curve fitting) or temporal smoothing at a post-processing step. Therefore we consider the median error as a very relevant measure of accuracy, besides the common mean error (Table 2).

For the task of localization, we also consider evaluating the accuracy of the vehicle’s orientation angle prediction (Table 2). Note that in our case, such orientation is also computed using an image segmentation approach. In particular, the vehicle’s heading is predicted using a half-dot output (the half-dot is in essence a half-circle), such that the real-valued orientation of the half-circle, gives the continuous angle of the pose. More specifically, the angle is computed as the orientation of the vector joining the center of mass of the localization dot (circle), with the center of mass of the half-dot (half-circle). Therefore the orientation could be arbitrarily precise.

**Planned experimental analysis of navigation:** Next, we perform experiments on visual navigation (Section 6.3). One of the key goals of these experiments is to show that our system is at least as accurate as top methods in the literature on the task of steering angle prediction in autonomous driving—which is one of the main tasks in such literature.

Note that the top published methods are usually not trained to predict steering angle for several seconds in advance. They are all focusing on instantaneous steering angle estimation. Our approach is unique, in the sense that we predict trajectories for the next seven seconds, which gives us an advantage in being able to plan ahead the vehicle’s next movements. Therefore, in order to have a fair comparison to other competing methods we have used their published code and trained them to predict for different intervals of time, up to seven seconds in the future. Then we compared their accuracy in steering angle prediction to ours for different future time intervals, up to seven seconds (Table 3).

Trajectories also give us speed information since they are functions of time. Therefore, once predictions are made for different moments in time in the future, we can also estimate the vehicle’s speed and acceleration. This fact is important, since we could better plan in advance, when driving, the speed of the vehicle, acceleration and braking, not just its steering angle. Consequently, we compared all the methods on speed prediction as well, after training them separately for each time interval ahead, up to seven seconds (Table 3).

**Figure 6:**
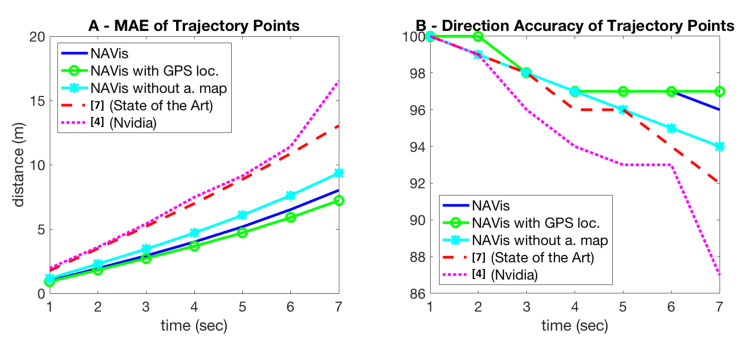
(**A**) Mean average error in seconds (sec) of time-trajectories points (lower is better). (**B**) the accuracy of direction in intersections for trajectory points (higher is better). The percentage of positions predicted on the correct route, is mostly higher for our models.

Going further, trajectories also offer us a vehicle 2D path, which is a function of distance (not time), so we could also estimate the correctness of the trajectory with respect to this future 2D path. We provide two ways of evaluation the path of the trajectory (Figure 6). One is from a pure spatio-temporal point of view, in which we estimate the distance between the predicted point on the trajectory at a certain moment in time and the corresponding real point on the true (ground-truth) trajectory that should be taken. Note that we have access to the real driver trajectory recorded on the test set for evaluation, which is considered as ground truth.

The other way of evaluating a trajectory is relative to how correct it is in terms of the higher-level task of navigation: how many times the predicted trajectory is close to the correct route in an intersection with several potential trajectories along different exit and entrance routes. This evaluation is harder to perform and we do it as follows: for a given predicted point on the trajectory, we consider it correct if the closest point to it, on a real road on the analytical map, is on the correct route that follows the correct directions (as computed using the analtical map and Dijkstra’s algorithm). This means that out of all directions, the predicted point on a trajectory indicates the correct direction if and only if the closest route on the actual road is the correct one (Figure 6).

For Section 6.2 and Section 6.3, we train our models on 89.72% of the first-phase dataset and test it on the remaining videos of approx. 2h total length. There are 12 continuous video sequences in the test set, distributed uniformly on the map.

**Planned experimental analysis of changing environmental conditions:** A very important dimension of experimental analysis in visual based localization and navigation must be the effect of environment changes, which could be short-term (due to weather and other temporary conditions) or long-term (changes in scene structure, road structure, city architecture etc.). While there are not many published works that study this aspect, we want to make sure that our system is able to adapt to such changes and perform well in terms of both localization and navigation in such cases of significant environmental changes. We present these experiments, considering different kinds of long-term and short-term changes in the third part of the experimental analysis (Section 6.4. We study and adapt the system for severe environmental changes on the data collected in the second stage, 70.1% of it being used for further training and the remaining 29.9% for testing. The first analyzed factor is the passing of a long time (of 14 months) between the two data collection phases. The other conditions, such as luminosity variations, sun flare, rain, and fog, are generated using realistic, good quality vision techniques (Table 4).

### 6.2. Experimental Results on Localization

We evaluate the response rate of the network (how often it outputs a dot), as well as the mean and the median errors of location and orientation (see Table 2). For the segmentation approach, we only take into consideration predicted dots with an area between 25% and 175% of the perfect’s dot area of 15px radius.

We compare several recent state of the art methods with different variants of our approach. In particular we considered the very influential state of the art methods [14,15] by Kendall et al. Both treat localization as a regression problem. The seminal Posenet paper [14] introduced localization as regression using a convolutional neural net for real-time 6-DOF pose estimation, while the method in [15] introduced a novel geometric loss in combination with deep learning for improved performance. We also compared to the recent work of Marcu et al, which is the first to formulate the localization task as segmentation and various versions of the current system (LOVis-2DOF, LOVis, LOVis-reg and LOVis-F), as follows: LOVis-2DOF is the basic localization as segmentation system presented here, without the orientation predicted as the half-circle segmentation; LOVis is the full deep net architecture with both localization (as full circle/dot) and orientation (as half circle/dot) prediction; LOVis-reg has almost the same deep net architecture as LOVis, with the only difference being that the orientation output (not the localization) is considered as a regression problem, instead of the initial half-dot segmentation; LOVis-F is the most advanced version of our system, in which we first project a predicted location onto every analytical path segment on the map, then replace it with its projection on the closest segment. In this step, we also interpolate predicted poses, when there is no prediction dot. Thus we obtain results for all examples in the test set. Afterwards, we perform additional time smoothing (using a very small temporal window of 5 frames) of the position by locally fitting time polynomials.

Segmentation approaches for location prediction [12], LOVis-2DOF, LOVis-reg, LOVis, LOVis-F perform better than the regression ones [14,15] in terms of precision, evincing that segmentation formulation is superior. Also, the PoseNet method proposed in 2017 [15] outperforms the earlier one from 2015 [14], as expected. The proposed data augmentation (LOVis-2DOF) raises the response percentage of [12] setup with 3.72% and decreases the mean error with about 10 m (indicating a significantly lower number of outliers), as the median error remains the same. Predicting the orientation by regression together with the position by segmentation (LOVis-reg) is not a successful approach, as it decreases the performances of LOVis-2DOF for position, and the results for orientation are worse than in the case of the full regression methods.

By contrast, when the network is modified to perform both position and orientation prediction by segmentation (LOVis), the results for the position task alone rise, so the response rate is 1.33 higher, and the errors are slightly lower. The performance also improves when we project the predicted locations on the analytical map segments (LOVis-F).

### 6.3. Experimental Results on Navigation

From the prediction of trajectories, we can evaluate the network’s performances at both steering and following route directions. Since long-time movement prediction is an unexplored subject in current autonomous driving research, we cannot evaluate our method directly against other solutions. In the following experiments, we modify a baseline [4] end-to-end steering model (by Nvidia) and the state-of-the-art one [7] to output steering commands for seven time-steps instead of just one. As the model in [7] has a separate branch for visual navigational information, we provide it the same two concatenated map images, like in the case of our model. The baseline model form Nvidia, however, learns only from single frames, according to its design.

First, we evaluate the steering performances like in the related literature, but over multiple time steps in the future (Table 3). The average control commands of speed and angle on seven time intervals of one-second length are derived from the trajectories outputted by our navigation net. In Table 3 we present experimental comparisons with the steering methods w.r.t. steering angle and speed performances. For the speed prediction, our model achieves significantly lower errors than both steering ones. The angle errors of our method and the one in [7] evolve tightly together over time, whereas the ones for [4] are higher.

**Figure 7:**
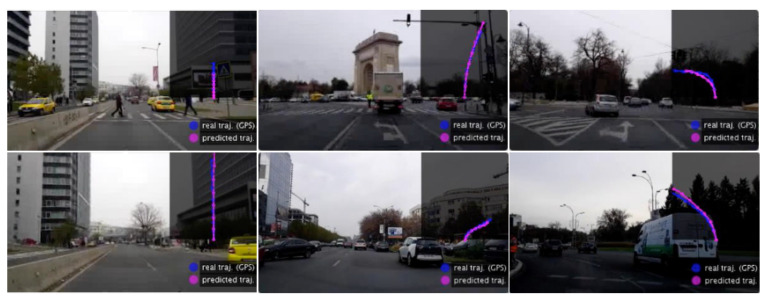
The trajectory predicted by the model in various traffic situations: stopping before a crosswalk (top **left**), accelerating when the road is clear (bottom **left**), before entering a roundabout (top **middle**), beginning to turn right (bottom **middle**), just before turning left (top **right**) and keeping left steer in a roundabout (bottom **right**). Notice how the trajectory, as a function of time also provides speed and acceleration information, not just spatial directions along a path. More specifically, the length of the trajectory indicates distance travelled per time. Thus, the spacing between the predicted locations in the next seven seconds are directly proportional to speed at those moments in time. The more distant the points, the faster the vehicle moves. Also, the variations in such spacing is directly related to acceleration, as the first derivative of speed with respect to time.

To better evaluate the navigation capabilities of the competing models, we introduce a direction performance metric, for when the vehicle is an intersection, and different paths may be correct depending on the destination. At a given moment in time, each approach (either based on trajectory prediction or direct multi-step steering and speed prediction) estimates the location of the vehicle for each of the next time steps. We are interested in evaluating if the locations are close to the road segment that belongs to the correct route. By projecting every predicted position on the map segments, we can immediately estimate how often the right path is the closest to the evaluated location among several possible ones. In Figure 6B, we show the accuracy rates of directions (the percentage of times the paths are correct when in an intersection), at each second, for our approach vs. the steering ones. We notice a drop in performance as the time step increases, with our net having the lowest rate of decrease compared to [4,7]. (which has by far the worst performances). We also compare setups similar to NAVis, to better understand the impact of using the analytical map and the pose prediction (vs. true GPS signal) in the navigation performance. As expected, the accuracy drops a bit when navigation is learned without using the analytical map at the input. Also, the performance improves when using the GPS signal. All variants of our models (with trajectory output) have results similar or better than the ones of the steering models. In Figure 7, we present a few qualitative trajectory results during test time, which illustrate the ability of our model to handle relatively complex situations, agreeing with the ground truth.

### 6.4. Experimental Results on Changing Environmental Conditions

**Figure 8:**
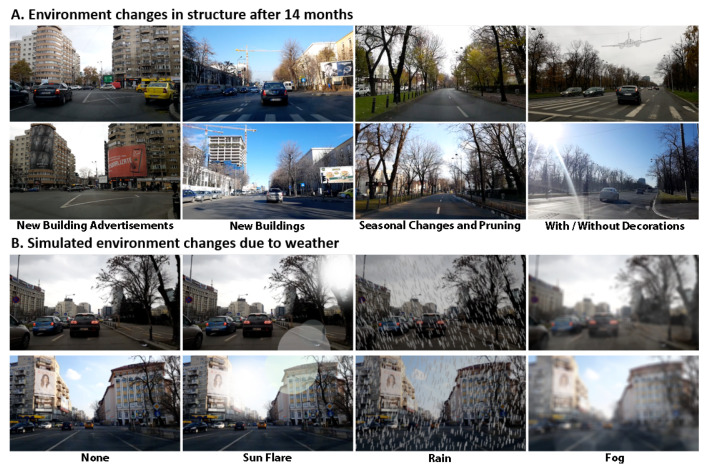
Examples of changes in environmental conditions. (**A**) Long-term structural changes. (**B**) Short-term changes in weather (artificially simulated).

The conditions in our first-phase dataset vary considerably due to rain, bad traffic, motion blur, and bad weather. However, some of these changes only occur on a small set of frames, having a low impact on networks’ training and performances. We study and adapt the system for severe environmental changes on the data collected in the second stage, 70.1% of it being used for further training and the remaining 29.9% for testing. The first analyzed factor is the passing of a long time (of 14 months) between the two data collection phases. The other conditions, namely luminosity variations, sun flare, rain, and fog, are generated using vision techniques (Figure 8B).

Time is a critical factor affecting the robustness of visual localization systems, as shown in [29]. In the long-term, both humans and nature change the environment’s appearance, such that visual cues learned by the network are no longer present (Figure 8A). For this reason, we firstly fine-tuned the network’s weights on the new raw training data. We did not start the training from the best-performing weights, as the LOVis net could not exit its local minimum, but from the ones in an earlier moment of the training. By receiving a significantly lower percentage of training examples, the network was able to slightly exceed its previous performances on the new test data (Table 4 row LOVis, column None). Moreover, LOVis seems reasonably robust to the high variations introduced by the generated conditions.

We fine-tune LOVis-W in the same way as LOVis, but its training data is augmented by applying all four environmental changes to the frames, resulting in a five-time larger training set. It raises the performances on all studied conditions, making them constant, at values very close to the ones on the noiseless data. Training with noise, acts as a regularization factor of the objective function [42], increasing generalization, to achieves slightly better results on the raw data (for the response rate and the mean error metrics; the median error remains constant).

**Figure 9:**
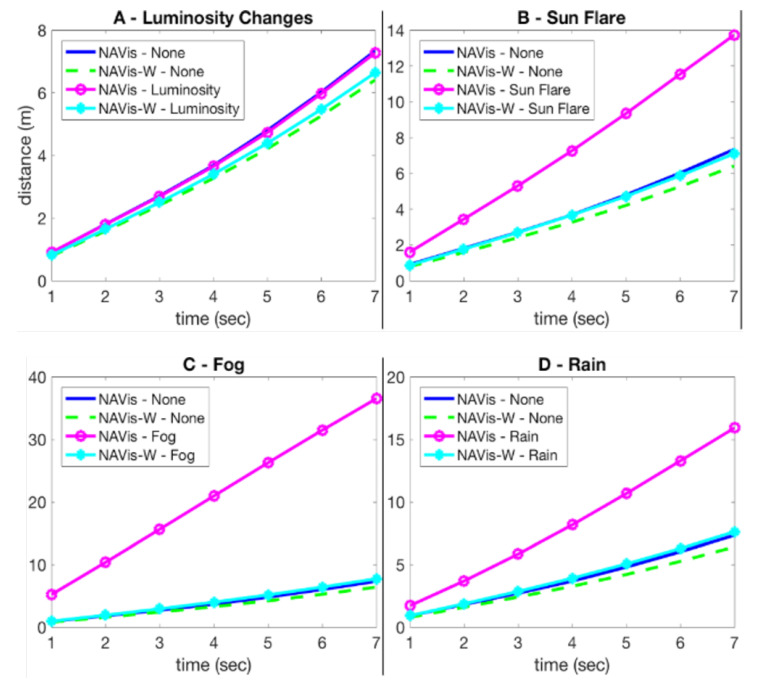
MAE (Mean Average Error) measured in seconds (sec) of predicted trajectories on changing environmental conditions.

As the correct trajectory depends mostly on the traffic and the infrastructure (factors independent of long-time), the navigation net matches its first performances on the new test set with no additional training (NAVis—None, Figure 9). However, its performances drop severely on unfavorable conditions such as sun flare effect, rain, and especially fog (NAVis—Sun Flare, Fog, Rain, Figure 9B–D). So, its robustness to the studied factors is opposite than in the case of the localization net. NAVis-W’s weights are fine-tuned on the data augmented with the environment-specific noise. Its errors on the changing conditions are significantly lower than the ones of NAVis (cyan vs. magenta lines, Figure 9). The fact that the performances are very close to the ones on the raw data demonstrates the robustness of NAVis-W to the simulated environmental conditions.

## 7. Open Research Questions and Future Work

Starting from our Driven by Vision system presented here, there are still a number of important open research questions remaining. They open the door for exciting new research directions, which we will consider in our future work. Below we enumerate and discuss such questions in detail, as next steps of study following our proposed approach:

1. Probably the first research question that should be addressed next is how to best combine the visual localization and navigation approach with the more traditional GPS-based one? While the GPS-based navigation is more robust and works in almost any driving condition, it also has limitations and can often fail or have delays, especially in scenes cluttered with tall buildings, trees and other large structures. On the other hand, the visual system can fail when the scenes are not distinctive enough, very similar to other scenes from other places, when the driver view is obstructed by traffic, or when a completely new scene is visited (by the system) for the first time. In such cases, the GPS could still be working fine. At the same time, the visual-based navigation system, when it works well, it is expected to be real-time and faster than the GPS-based one. The two approaches, visual and GPS-based, are definitely complementary and can help each other for a more robust and accurate prediction. Thus, they offer an excellent open research question and next topic of work.
2. The second open research question is related to the scalability of the learning-based, visual navigation system. What is the best way to collect training data from many users and integrate it into a single coherent database, in order to learn efficiently a single unified system. The scalability problem becomes interesting from many reasons, including aspects of speed, computation and memory requirements, but also the aspect of reusing resources and data. We expect that many users will often pass through the same common streets and scenes, while other more secluded scenes will be less covered by drivers. In such cases, an intelligent and efficient balancing system is needed in order to give different priorities to data covering new territory vs data covering well-known, already mapped areas. Such a novel intelligent system, for handling scalability of learning, is needed in order to integrate all these cases in the most optimal way for robust and well-balanced training.
3. The third important research question we propose, is how to best provide visual navigation information to drivers, since, in the visual approach to navigation, vision and navigation are tightly connected. What is the best way to offer visual assistance to a driver for optimal results and how can this aspect be best evaluated and improved? While the topic falls in the realm of human-computer interaction (HCI), it is definitely an important one for future research, in order to get the most out of visual navigation.
4. Another important research question comes from the ability of the system to adapt to structural and weather changes. We have proved in our experiments that this adaptation can work well. However, we have tested only over a period of 14 months, not longer. Over much longer periods, the structural and environmental changes at a given location are expected to be much more significant. It would be interesting to see what is the best way for the system to start forgetting the old road structures and scenes and adapt to the new ones as they change over time. One particularly interesting aspect here is the fact that some streets and scenes change more than others, over time.
5. Other interesting open research questions, relevant for visual-based navigation, include the following: what are the regions, categories of objects or features in the scene that are best for localization. How about the features, objects and classes in the scene that are best for navigation? Is there a real advantage in using some other high-level auxiliary tasks (e.g., semantic segmentation of the scene, prediction of depth and 3D structure, detection of moving objects and other object categories in the scene) in order to improve localization and navigation? Also, is there a better way of using spatio-temporal processing? So far we have used only a relatively small temporal window of frames around the current time. It is not yet clear how much temporal processing is required, what is the optimal period of past time to be considered and what deep network models (e.g., recurrent in space and time) are most appropriate for visual navigation. In our experiments, the localization task seems to work fine without having to consider significant time processing. At the same time, we do expect the higher-level problem of navigation to be better suited for more sophisticated temporal processing and combination with higher-level auxiliary tasks.

## 8. Conclusions

We present the first deep-learning, end-to-end system that learns to self-locate and to navigate towards a planned destination by relying only on visual information. The system is low-cost and requires only a regular car and smartphone during the automatic data acquisition, annotation, and testing. We also present a mathematical method for automatically constructing an analytical road map from the collected data and introduce a relatively large dataset, collected and mapped in this manner. We present novel solutions to robust localization by image segmentation and trajectory prediction and demonstrate state of the art performance in all our tests. We further show how our approach can adapt to short and long term changes in weather and the environment over time. We put it all together into a complete high-performance system that can perform all necessary steps, with minimal human intervention for learning visual navigation. Our approach is scalable and competitive, and for these reasons, it has the potential to be a solid contribution to research in autonomous driving and navigation assistance. We conclude our work by presenting a list of relevant open research questions and future directions, which follow naturally from the proposed approach, opening the door for our next steps of research.

## Figures and Tables

**Table 1:** Comparison of real-world driving datasets for imitation learning.

Dataset	Driving Time (h)	GPS/IMU	Route Planner	Autonomous Route Planner	CAN Bus Reader	Lidar	Accessible Setup
BDDV [5]	10k	Yes	No	No	No	No	Yes
Cityscapes [34]	<100	Yes	No	No	No	No	No
Comma.ai [35]	7.3	Yes	No	No	Yes	No	No
Drive360 [7]	60	Yes	Yes	No	Yes	No	No
KITTI [33]	<1	Yes	No	No	No	Yes	No
Oxford [36]	214	Yes	No	No	No	Yes	No
Udacity [37]	10	Yes	No	No	Yes	No	No
UED (ours)	21	Yes	Yes	Yes	No	No	Yes

**Table 2:** Results for 3-DOF pose prediction. Note how the segmentation formulation (Seg) is superior to the regression one (Reg) for both position and orientation learning. The best performances are shown in bold.

	Position				Orientation			
Method	Type	Response	Mean	Median	Type	Response	Mean	Median
[14]	Reg	**100%**	58.09 m	17.75 m	Reg	**100%**	7.81°	2.41°
[15]	Reg	**100%**	50.84 m	15.39 m	Reg	**100%**	7.33°	1.88°
[12]	Seg	91.00%	27.36 m	11.44 m	-	-	-	-
LOVis-2DOF	Seg	94.72%	17.31 m	11.55 m	-	-	-	-
LOVis-reg	Seg	92.62%	26.95 m	13.95 m	Reg	**100%**	9.92°	4.41°
LOVis	Seg	96.35%	16.89 m	11.18 m	Seg	96.08%	**3.65°**	1.43°
LOVis-F	Seg	**100%**	**16.05 m**	**10.90 m**	Seg	**100%**	3.73°	**0.67°**

**Table 3:** Mean errors for speed and steering angle (lower is better). The best performances are shown in bold.

	MAE Speed (m/s)							MAE Steering Angle (°)						
Method	1 s	2 s	3 s	4 s	5 s	6 s	7 s	1 s	2 s	3 s	4 s	5 s	6 s	7 s
[4]	1.9	1.91	1.99	1.94	1.96	1.95	2.33	1.01	1.61	2.09	2.65	3.14	3.9	5.48
[7]	1.76	1.7	1.68	1.68	1.69	1.72	1.76	0.91	1.32	1.74	2.05	2.39	2.95	4.3
NAVis	**0.94**	**0.92**	**0.91**	**0.92**	**0.94**	**0.98**	**1.03**	**0.84**	**1.26**	**1.68**	**2**	**2.36**	**2.91**	**4.24**

**Table 4:** Results for 3-DOF pose prediction on various environmental conditions. The best performances are shown in bold.

**Position: response (%), mean (meters), median (meters)**															
	None			Luminosity			Sun Flare			Fog			Rain		
LOVis	97.9	14.7	**11.2**	87.8	26.9	12.3	86.5	27.1	12.6	78.5	31	12.3	74.2	22.7	14
LOVis-W	98.3	13.6	**11.2**	97.7	14.3	11.6	97.5	14.9	11.4	97.6	14.6	11.8	97.8	14.2	11.5
LOVis-WF	**100**	**13**	**11.2**	**100**	**13.2**	**11.5**	**100**	**13.2**	**11.3**	**100**	**13.4**	**11.7**	**100**	**13.3**	**11.3**
**Orientation: response (%), mean (degrees), median (degrees)**															
	None			Luminosity			Sun Flare			Fog			Rain		
LOVis	97.3	2.59	1.15	87.1	3.81	1.52	85.5	4.48	1.47	77.5	5.08	1.57	73.3	3.86	1.66
LOVis-W	98.2	**2.23**	**1.06**	97.5	**2.44**	**1.18**	97.2	**2.58**	**1.26**	97.5	**2.52**	**1.21**	97.6	**2.41**	**1.24**
LOVis-WF	**100**	2.28	1.07	**100**	2.51	1.19	**100**	2.68	1.27	**100**	2.56	1.22	**100**	2.46	1.25

## Data Availability

The data and code used for this article is publicly available on the Driven by Vision project website: https://sites.google.com/view/drivenbyvision/home.
